# Stages of Change in Physical Activity and Neighborhood Walkability Among Older Adults living in the Urban Setting

**DOI:** 10.1093/geroni/igab046.2364

**Published:** 2021-12-17

**Authors:** Jennifer Blackwood, Rie Suzuki, Noah Webster, Shailee Shah

**Affiliations:** 1 University of Michigan--Flint, Michigan, United States; 2 University of Michigan-Flint, Flint, Michigan, United States; 3 University of Michigan, Ann Arbor, Michigan, United States; 4 University of Michigan-Flint, University of Michigan-Flint, Michigan, United States

## Abstract

Insufficient physical activity (PA) is considered an independent risk factor for chronic diseases. Although older adults living in lower-income areas often experience obstacles to walking locally, few studies have compared their walking experiences and the degree of readiness to change on engaging in PA. The purpose of this study was to compare perceptions of neighborhood walkability by the stages of change among older adults living in a lower-income community. Participants were recruited in 2018 at a regional health clinic in Flint, MI. To be eligible, participants had to be over 65 years old and Flint residents. Of the 132 participants, the mean age was 69.74 (SD=5.00) years old. The majority of respondents were female (66%); African American (77%); single, divorced, or widowed (75%); and educated below a GED level (84%). The results showed that older adults at the pre-contemplation/contemplation stage (PC/C) were less likely to perceive the availability of sidewalks on most streets and more likely to complain about much traffic along the street than those at the action/maintenance stage (A/M) (p < 0.05). After controlling for covariates, multiple regression analysis showed that those at PC/C were less likely to state that their neighborhoods were accessible (β = .17*) and to perceive the presence of walking hazard (e.g., lack of sidewalks) (β = -.17*). Those who engaged in PA less than 30 minutes per day perceived the neighborhoods were accessible (β = .23*). Findings suggest that it is essential to develop friendly support systems and accommodations to encourage walking in lower-income communities.

